# LF-NMR/MRI Determination of Different 6-Benzylaminopurine Concentrations and Their Effects on Soybean Moisture

**DOI:** 10.3389/fpls.2022.885804

**Published:** 2022-04-18

**Authors:** Ying Gu, Yi Chen, Xia Yue, Peng Xiong, Dayu Pan, Ping Song, Bin Luo

**Affiliations:** ^1^College of Information and Electrical Engineering, Shenyang Agricultural University, Shenyang, China; ^2^Research Center of Intelligent Equipment, Beijing Academy of Agriculture and Forestry Sciences, Beijing, China

**Keywords:** 6-benzylaminopurine, magnetic resonance imaging, nuclear magnetic resonance, water dynamics, principal component analysis

## Abstract

In this study, we aimed to clarify the distribution and dynamics of water in the Xudou 20 soybean cultivar post-germination after culturing plants with various concentrations of 6-benzylaminopurine (6-BA). Low-field nuclear magnetic resonance and magnetic resonance imaging (LF-NMR/MRI), as well as principal component analysis (PCA), were used for the investigation. Results showed that low concentrations of 6-BA promoted soybean germination and high concentrations inhibited soybean germination, with 5 mg/l of 6-BA producing the most optimal conditions for growth. Moreover, the *T*_22_ determination of weakly bound water increased with increasing 6-BA concentration, and the PCA effectively distinguished soybeans cultured at different 6-BA concentrations. This study provides a method for the rapid detection of 6-BA concentration in bean sprouts and provides theoretical support and bean sprout quality assessment.

**Graphical Abstract:**
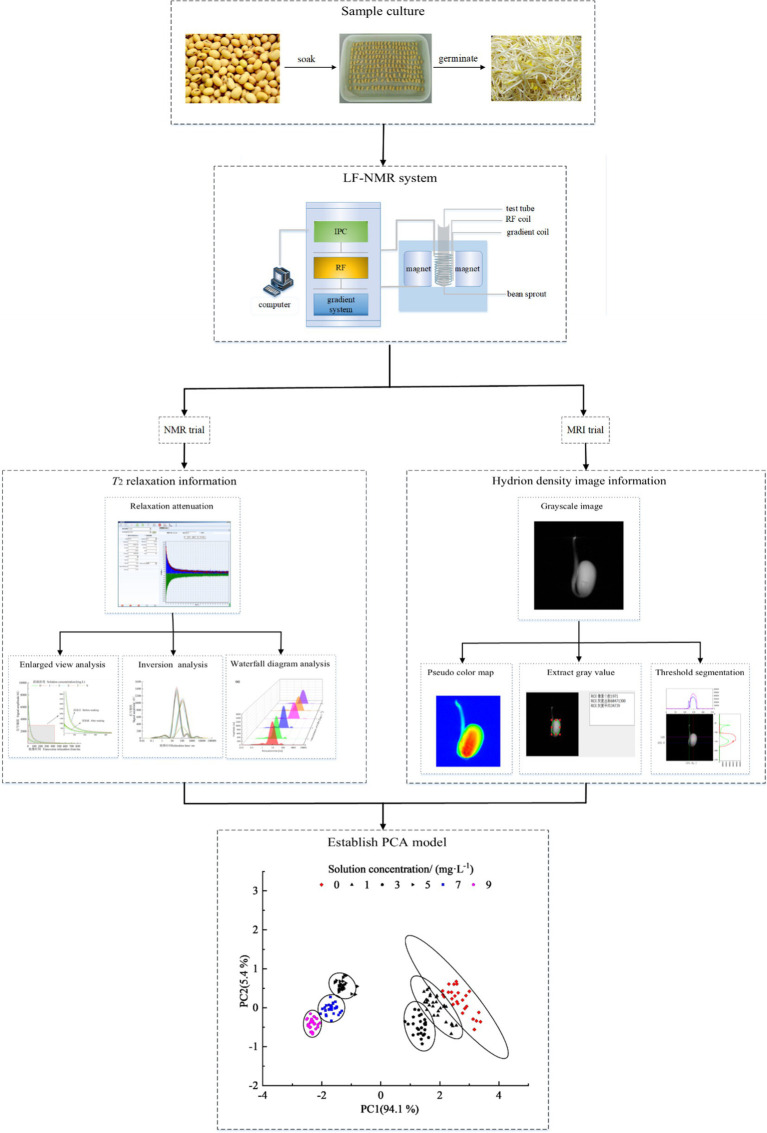


## Introduction

Soybean is cultivated worldwide, with a wide planting range and a large area of China. Soybeans are rich in protein and provide eight essential amino acids (Jin, 2020). In addition, they contain a biological coagulant that can promote immunity, dietary fibers that can improve the function of digestive organs, and peptides that reduce blood glucose and blood pressure, promoting weight loss. However, their nutritional value is limited by anti-nutritional factors such as trypsin inhibitors as well as raffinose, which is not readily absorbed by the human body and can lead to abdominal distension. However, after the germination treatment, trypsin inhibitors in soybean are degraded, and the raffinose content also decreases (Urbano et al., 2005). Moreover, the post-germination soybean protein content is higher than before germination. In countries where beans are the main source of plant protein, such as Egypt and Pakistan, soybean germination is a common practice to improve the nutritional value of beans and eliminate unfavorable characteristics (Sathe et al., 1992).

6-BA is a synthetic white crystal powder that promotes cell division and induces tissue differentiation. It is often used to regulate plant growth and development. The effects of 6-BA are species-specific and influenced by concentrations and treatment lengths (Wang et al., 2010; Zhang et al., 2019). Although it is possible to increase crop yield using 6-BA, numerous illegal businesses or small workshops use excessive 6-BA amounts to shorten the sprouting cycle and improve the appearance and tenderness of bean sprouts. This poses a significant threat to consumers’ health. Japan classifies bean sprouts as “other vegetables” and stipulates that the maximum residue limit of 6-BA of other vegetable agricultural chemicals is <0.5 mg/kg. Through experiments, we found that 0 mg/l ≤ 6-BA ≤ 3 mg/l is an appropriate concentration, and the residue is less than 0.5 mg/kg, which satisfies the criterion above; 6-BA >3 mg/l is excessive, and the residue is greater than 0.5 mg/kg. Conventional methods for detecting the 6-BA composition of bean sprouts include rapid solvent extraction pretreatment technology and surface-enhanced Raman Spectroscopy (Zhang et al., 2012), high-performance liquid chromatography (Lin et al., 2013; Tang, 2021), resonance Rayleigh scattering, and fluorescence. Although these methods are characterized by relatively high accuracy and sensitivity, they are associated with several disadvantages, such as ease of sample destruction, long detection time, use of toxic solvents, and complex sample purification. Water absorption during the process of soybean soaking results in the hydration of the whole soybean and is necessary for the initiation and termination of soybean germination (Yue et al., 2020). The morphological structure of beans is complex. To date, there is no clear study on the transmission path of water into soybeans. Several researchers believe that the seed coat is the main factor influencing water dynamics in bean water systems because the external water must overcome the barrier of the seed coat to penetrate the soybean (Chachalis and Smith, 2001). In contrast, other researchers have reported that external water predominately enters the soybean through the seed navel (Manning and Van Staden, 1985; McDonald et al., 1988). Experimental evidence shows that seed holes are the main channel for external water to enter and leave the soybean (Kulik and Yaklich, 1991). In order to study the process of external water entering soybean during soybean soaking and the effect of different 6-BA concentrations on soybean water content, experiments were performed to detect changes in soybean hydrogen proton content to assess the water distribution and dynamics in soybean seeds.

As an emerging technology, LF-NMR/MRI has been widely used in energy and geotechnical studies (Ouyang, 2017; Dou, 2019), life sciences (Jiang et al., 2010), industrial nuclear magnetic analyses (Kirtil and Oztop, 2016; Hou et al., 2021; Liu et al., 2021), food (Lin et al., 2016; Zhu, 2016; Zhu et al., 2020), and agriculture (Song et al., 2018). The advantages associated with this method include non-destruction of samples, environmental protection, as well as rapid and highly accurate detection. Because of these characteristics, this method is also widely used to detect adulterated honey, oil, and meat products (Ribeiro et al., 2014; Li et al., 2018). The kinetic information between the sample molecules and molecules is detected by LF-NMR, and the data are collected by using an appropriate pulse sequence. The parameters such as relaxation time, relaxation peak position, and relaxation peak area quantitatively reflect the ability of proton motion in this state. LF-MRI uses the hydrogen atomic nucleus with spin characteristics to stop receiving radio frequency pulse excitation in a special magnetic field, and the resonance signal generated in the process of releasing energy is acquired by an external receiver, and processed by an electronic computer to obtain an image. Moisture plays an important role in the process of seed germination, so LF-NMR/MRI can be used to detect the changes of water in various phases and other growth information during the process of seed germination to analyze the growth state of seeds.

In this study, the soybean cultivar Xudou 20 was cultured in 6-BA solutions at concentrations of 0, 1, 3, 5, 7, and 9 mg/l to explore the effects of different 6-BA concentrations on the water distribution and dynamics in germinating seeds. Proton density gray maps of the plant sagittal plane were collected every 1 h, and the hydrogen spectrum was obtained every 12 h. At the same time, the horizontal axis diameter, vertical axis diameter, and fresh soybean quality data of all the test samples were measured. Moreover, PCA analysis was used to distinguish between soybean sprouts cultured in the 6-BA solution at different concentrations to provide theoretical support and solutions for soybean production and bean sprout quality detection.

## Materials and Methods

### Samples

Xudou 20 has a high yield and strong stress resistance, suitable for sowing in many places in China. Therefore, Xudou 20 was selected as the experimental object. Xudou 20 was purchased from a general agricultural materials market. It is bred by cross-breeding Xudou 9 as the female parent and Xudou 10 as the male parent (Wang et al., 2015; Wang, 2019).

### LF-NMR Measurements

An LF-NMR/MRI analyzer NMI20-015V-I (Newmag Co., Ltd. Suzhou, China) was used for the LF-NMR/MRI measurements. The characteristics of this instrument are as follows: magnetic field intensity, (0.5 ± 0.08) T; radio frequency pulses (RF), 18 MHz; magnet temperature, 32°C; probe coil diameter, 15 mm. Hydrogen spectrum trial transverse relaxation times (*T*_2_) were measured using the Carr-Purcell-Meiboom-Gill (CPMG) sequence. The parameters of CPMG were set as follows: corresponding resonances frequency (SF) for protons = 21 MHz; spectral width (SW) = 200 kHz; echo time (TE) = 0.25 μs; pulse widths at 90° (P1) and 180° (P2), 16 and 36 μs, respectively; waiting time (TW) = 2000 μs; radio frequency delay time (RFD) = 0.02 μs; analog gain (RG1) = 20 db; digital gain (DGR1) = 3; and number of echoes (NECH) = 3,000.

### LF-MRI Measurements

The parameters of the imaging experiment were set as follows: in the visual field adjustment area, the field of view (FOV) read, and FOV phase were 60.0 mm collectively, and the offset read (OR) was 0 mm. In the layer selection adjustment area, the offset slice was −1.6 mm; the slice was set to 1; the slice width was 10 mm; the layer spacing was 2.1 mm; the deflection angle *β* was 90°; the other deflection angles were 0°. Moreover, TE was set to 5.885 ms, the repeat sampling time (TR) was 300 ms; the number of repeated samples was set to 32. The sagittal image information of the experimental samples was collected, and each sampling time was 30 min.

### Samples Preparation

Prior to the experiment, 1, 3, 5, 7, and 9 mg of the 6-BA crystal sample (≥99%, Shanghai Sigma-Aldrich Trading Co., Ltd) were weighed out. Then, the 6-BA crystals were placed in a 50-ml beaker with 2 ml of NaOH (0.5 mol/l, Guangzhou Hewei Pharmaceutical Technology Co., Ltd.) solution. The beaker was placed into a 60°C water bath and stirred with a glass stirring rod until the 6-BA crystal was completely dissolved. Finally, the dissolved 6-BA crystal was used to make 1, 3, 5, 7, and 9 mg/l solutions and stored in a 1 L volumetric flask for subsequent experimental use. 6-BA solutions with six concentration gradients of 0, 1, 3, 5, 7, and 9 mg/l were used in this experiment, and deionized water (0 mg/l) was used as a control. After analysis through multiple experiments, in this article, 6-BA with a concentration higher than 5 mg/l (excluding 5 mg/l) is high concentration solution, and less than 5 mg/l (including 5 mg/l) of 6-BA is low concentration solution.

During the experiment, soybeans with similar sizes and shapes, no surface damage, and masses of approximately 0.25 ± 0.05 g were selected. A total of 120 soybeans were selected for experimental analysis. Twenty parallel samples were set for each concentration gradient in the LF-NMR spectrum experiment. The measurement was repeated three times for each sample, after which the average value was taken. Five parallel samples were randomly selected for each concentration gradient in the LF-MRI experiment, and three replicate measurements were conducted for each soybean. Soybeans were cultured in an intelligent artificial climate incubator (Zhejiang Tuopu Instrument Co., Ltd.) at a set temperature of 25°C and an RH of 45%. Before culturing, soybeans were sterilized with a NaClO (0.01%, Jiangbiao Detection Technology Co., Ltd.) solution for 15 min, rinsed 3–5 times with deionized water at a constant water temperature of 25°C ± 1°C, and soaked in a solution with a volume ratio of 1:3 (experimental sample:solution). The samples were soaked for 8 h, and the sagittal proton density gray map of the experimental samples was obtained every 1 h. Samples were incubated for 60 h without light, and the *T*_2_ relaxation time of the experiment was obtained every 12 h. Vernier calipers (Shengtaixin Technology Co., Ltd.) were used to measure the transverse and longitudinal axes diameters of all the experimental samples. According to the fresh soybean mass and transverse and longitudinal axis diameter data collected hourly during the soaking process, the growth rates of the soybeans before and after soaking were calculated according to Eqs. (1)–(3):


(1) $$m=\left(m_{2}-m_{1}\right)/m_{1}\times 100\%$$

(2) $$d=\left(d_{2}-d_{1}\right)/d_{1}\times 100\%$$

(3) $$r=\left(r_{2}-r_{1}\right)/r_{1}\times 100\%$$

where *m* is the fresh quality growth rate, *d* is the horizontal axis diameter growth rate, and *r* is the longitudinal axis diameter growth rate. In addition, subscripts 1 and 2 represent the corresponding parameters before and after soaking, respectively.

### Statistical Analysis

Niumag NMR Analysis Application software Ver4.0 (Niumag Co., Ltd., Suzhou, China) was used for data analysis and distributed exponential curve fitting. The continuous distribution of exponentials for the CPMG experiment is defined by Eq. (4):


(4) $$M\left(t\right)=\underset{i}{\sum }P_{i}\exp\left(-\frac{t}{T_{2i}}\right)+\varepsilon \left(t\right)$$

where *M(t)* is the residual magnetization at a given time *t* after applying the first radio frequency pulse; *P_i_* and *T*_2*i*_ are the spin–spin relaxation amplitude and time, respectively, of the *i^th^* component; and ε(t) is the residual error.

Although soybeans with similar characteristics were selected for the experiment, the quality of each soybean could not be guaranteed to be similar; thus, it was necessary to eliminate the influence of dimension on the experimental results. Therefore, after obtaining the *T*_2_ parameter, the data were normalized according to Eq. (5):


(5) $$\mathrm{Signal}\;\;\mathrm{per}\;\mathrm{mass}\;\mathrm{au}\cdot \mathrm{ms}=\frac{A_{2i}}{m}$$

where $A_{2i}$ is the corresponding water population (area ratio) of the *i*^th^ component, and *m* is the mass of the Xudou 20 sample. The signal per mass of the total water is the sum of each signal per mass component.

PCA and ANOVA were carried out for a set of observations using IBM SPSS (Statistical Package for the Social Sciences) statistics v. 23.0 (SPSS Inc., United States). All figures were plotted using Origin 2018 software (Microcal, United States). The obtained hydrogen proton density map was processed by pseudo-color using Niumag NMR image processing software (Niumag Co., Ltd.), and the region of interest (ROI) was extracted.

## Results and Discussion

### LF-MRI Result Analysis

**Figure 1:**
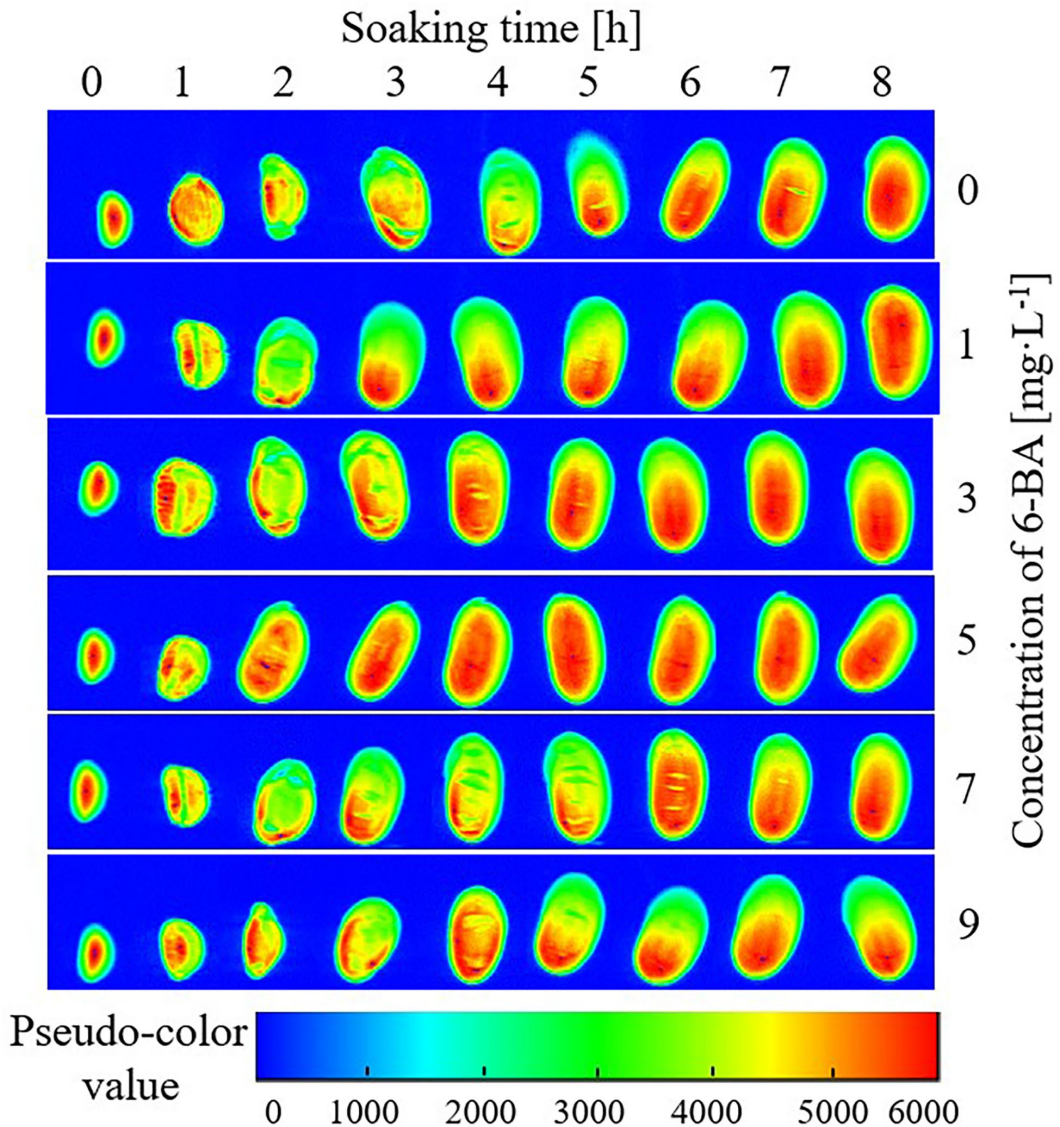
Pseudo-color map of continuous time points during soybean soaking in 6-BA solution with different concentrations. The larger the pseudo-color value, the greater the hydrogen proton density in the experimental sample.

To study the dynamics of water entering soybeans during the soaking process and determine the effect of different 6-BA concentrations on water entering the seeds, a pseudo-color map of the soybeans was constructed every 1 h for 8 h, as shown in Figure 1.

The pseudo-color map visually shows the distribution of water in the soybean. Red (high brightness) indicates a high hydrogen proton density and water holding capacity in the area, whereas blue (low brightness) indicates low hydrogen proton density and water holding capacity.

As shown in Figure 1, before soybean soaking, the water was concentrated in the center of the cotyledon (red in Figure 1), and the water gradually reduced in the center of the partial ion leaf. After soaking for 1 h, the volume of the soybean increased significantly, and water in the two cotyledons was evenly distributed after entering the soybean. After soaking for 2 h, the soybean seeds continued to absorb water and expand, and their morphology gradually changed. After soaking for 3 h, the morphology of the soybean did not change significantly. Except for the soybean soaked in 5 mg/l of 6-BA, the water was unevenly distributed in the experimental groups. After soaking for 4 h, the water content in the 3 mg/l and 9 mg/l 6-BA experimental groups significantly increased. Conversely, after soaking for 5 h, the water content of the experimental group with 9 mg/l 6-BA suddenly decreased. After soaking for 6–8 h, the water content and morphology of all experimental groups remained stable, and the water absorption reached the saturation value.

Water enters seeds with cell membranes of non-recovered functions and produces an imbalance of forces between local tissues from the swelling of cell constituents. Therefore, the time course of water uptake indicates that imbibition by dry seeds is, to some extent, accompanied by uneven swelling during the first entry of water. The signal intensity did not stop rising when the seed stopped expanding; water incorporation exceeded the expansion of the seed. This may be because inter- and intra-gas spaces of cells were diminished. Driving gases from small pores in cell constituents by replacement with water requires a large amount of free energy, a biological event. Another possibility is that the molecular structures of stored materials were rearranged to hold more water between the molecular structures, such as the transformation of starches from crystal to gel, and from gel to solution with release of cations. The conformational changes, or unfolding and refolding of macromolecular compounds, are biological processes (Kikuchi et al., 2006).

The trajectory of water entering the seed differs by species. For soybeans, water is believed to first pass through the seed coat, then through the micropyle and navel, and finally through the hypocotyl. Soybean imbibition reconstitutes the membrane structure, reactivates stored protein, and produces residual mRNA during soybean ripening before drying. The moisture and morphology of soybean changed during soaking. When 6-BA concentration in solution is higher than 5 mg/l leads to the denaturation of the soybean seed protein and produces a denser structure. In particular, soybean seeds may play a negative role in water retention in the initial soaking stage. As shown in Figure 1, with an increase in the 6-BA solution concentration, the water content in the soybean seeds gradually increased until saturation. However, when the 6-BA concentration exceeded 7 mg/l, the water content first increased and then decreased. When the concentration of the 6-BA solution was 5 mg/l, the water absorption of the soybean reached saturation fastest for the sample set, followed by 3 mg/l > 2 mg/l > 1 mg/l > 7 mg/l > 9 mg/l.

### Extract Region of Interest Value Results Analysis

**Figure 2:**
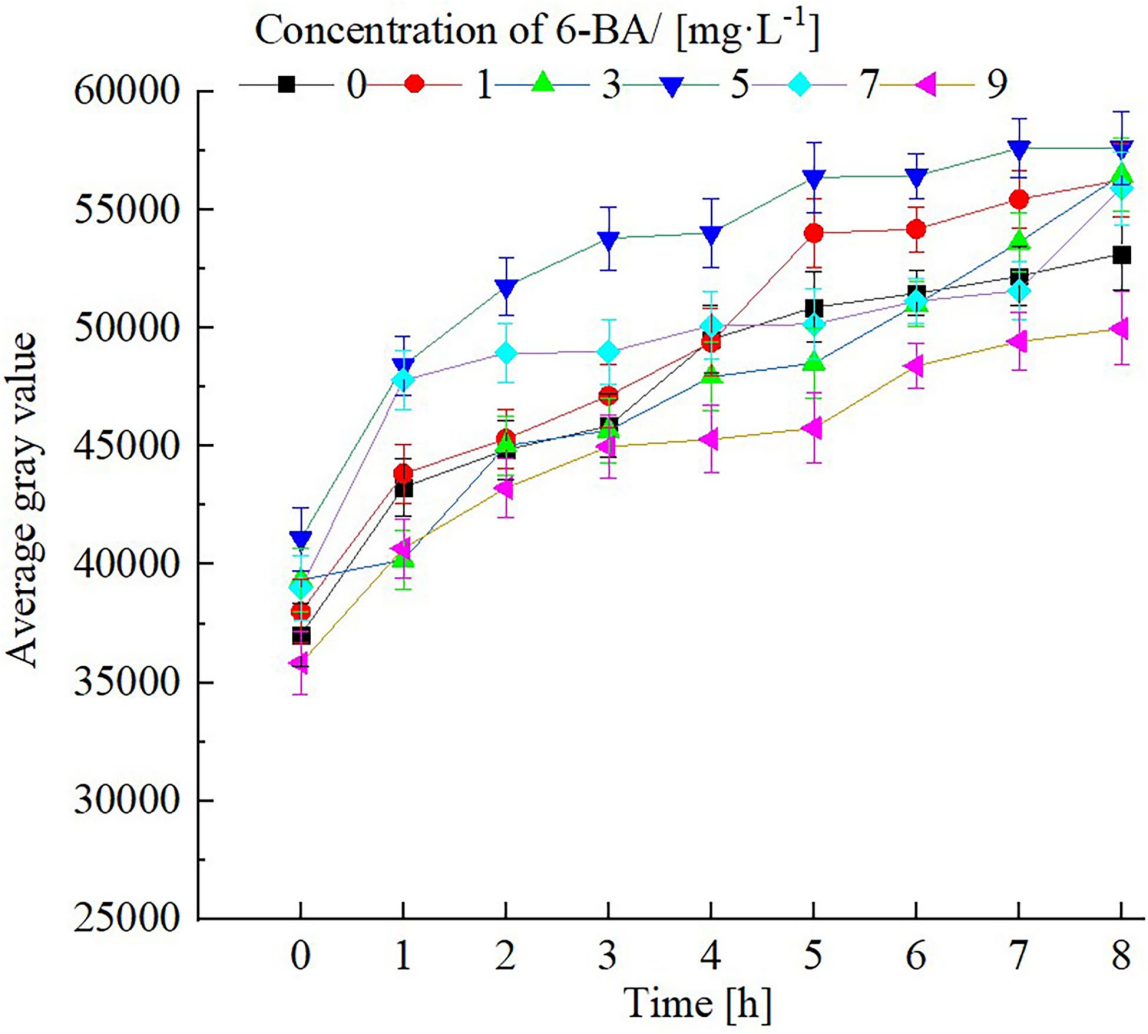
Average gray value variation curve for a region of interest of soybean during soaking in 6-BA solution with different concentrations.

We calculated the average gray value based on the extracted ROI from the gray image obtained from the NMR proton density image. We obtained the average gray value change curve of the test samples at intervals of 1 h during the immersion in solutions containing different 6-BA concentrations for 8 h. The corresponding calculation results are shown in Figure 2, where the horizontal mark represents the immersion period. The vertical coordinate represents the average gray value of the ROI.

The higher the average gray value, the higher the water content of the experimental sample during soaking. The water absorption of soybean during soaking depends on the water absorption capability of the protoplasm. This water absorption is unrelated to soybean metabolism; soybeans can absorb water in both dormant and vital states. The protoplast colloid of soybean makes the inactivated biological macromolecules stretch through water absorption, showing the original structure and characteristics.

During our experimental procedure, the average gray value of the test samples increased with increasing soaking time. After soaking for 6 h, the water absorption saturation curve of the soybean samples gradually reached a stable state. The average gray value of the soybeans soaked with 5 mg/l of 6-BA was significantly higher than that of soybeans soaked in other 6-BA concentrations; the average gray value of the soybean soaked in 9 mg/l 6-BA was the lowest. These results are consistent with the visual observation results of the pseudo-color map, which shows that an appropriate concentration of 6-BA can promote water absorption in soybeans and bean sprout growth. Conversely, high 6-BA concentrations (6-BA>5 mg/l) lead to the deterioration of the structure of the soybean and inhibition of water absorption, thus hindering the growth of soybean sprouts.

### Analysis of Growth Rate Results

**Figure 3:**
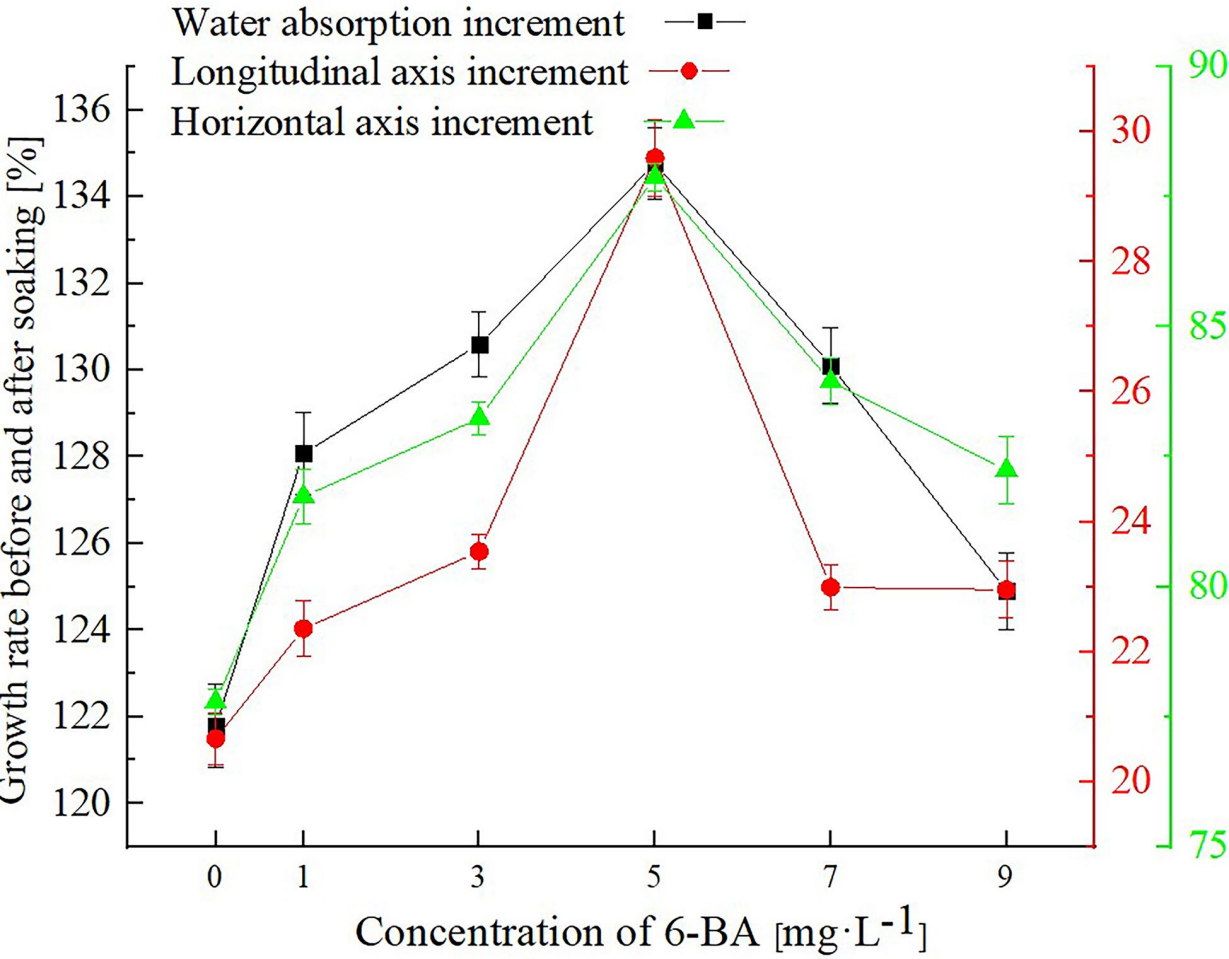
Variation curves for the fresh mass, and transverse and longitudinal axis diameter growth rates before and after immersion in various 6-BA solutions.

After calculating the results according to Eqs. (1)–(3), change curves were determined for the fresh mass as well as the transverse and longitudinal axis diameter growth rates of the soybeans before and after soaking in various 6-BA solutions, as shown in Figure 3.

Figure 3 is a multi-ordinate axis broken line diagram, in which the abscissa represents the concentrations of the 6-BA solution, the left axis of the ordinate represents the growth percentage of the soybean fresh mass before and after soaking, and the right axis of the ordinate represents the growth percentage of the soybean diameter before and after soaking. Using a concentration of 5 mg/l as the dividing point, the growth rate of the fresh mass and the transverse and longitudinal axis diameters of the soybean soaked in low 6-BA concentration solutions on the left increased with an increase in the solution concentration. Conversely, the soybean soaked in a high 6-BA concentration solution (6-BA>5 mg/l) showed an opposite trend, with a growth rate inversely proportional to the 6-BA concentration.

Notably, the primary factor influencing an increase in the quality and volume of the soybean is water. Therefore, it can be deduced that when the concentration of the 6-BA solution is 5 mg/l, the soaking efficiency of Xudou 20 soybeans improves. This observation is consistent with the NMR experimental analysis results.

### Analysis of Hydrogen Spectrum Results in LF-NMR Spectrum Experiment

**Figure 4:**
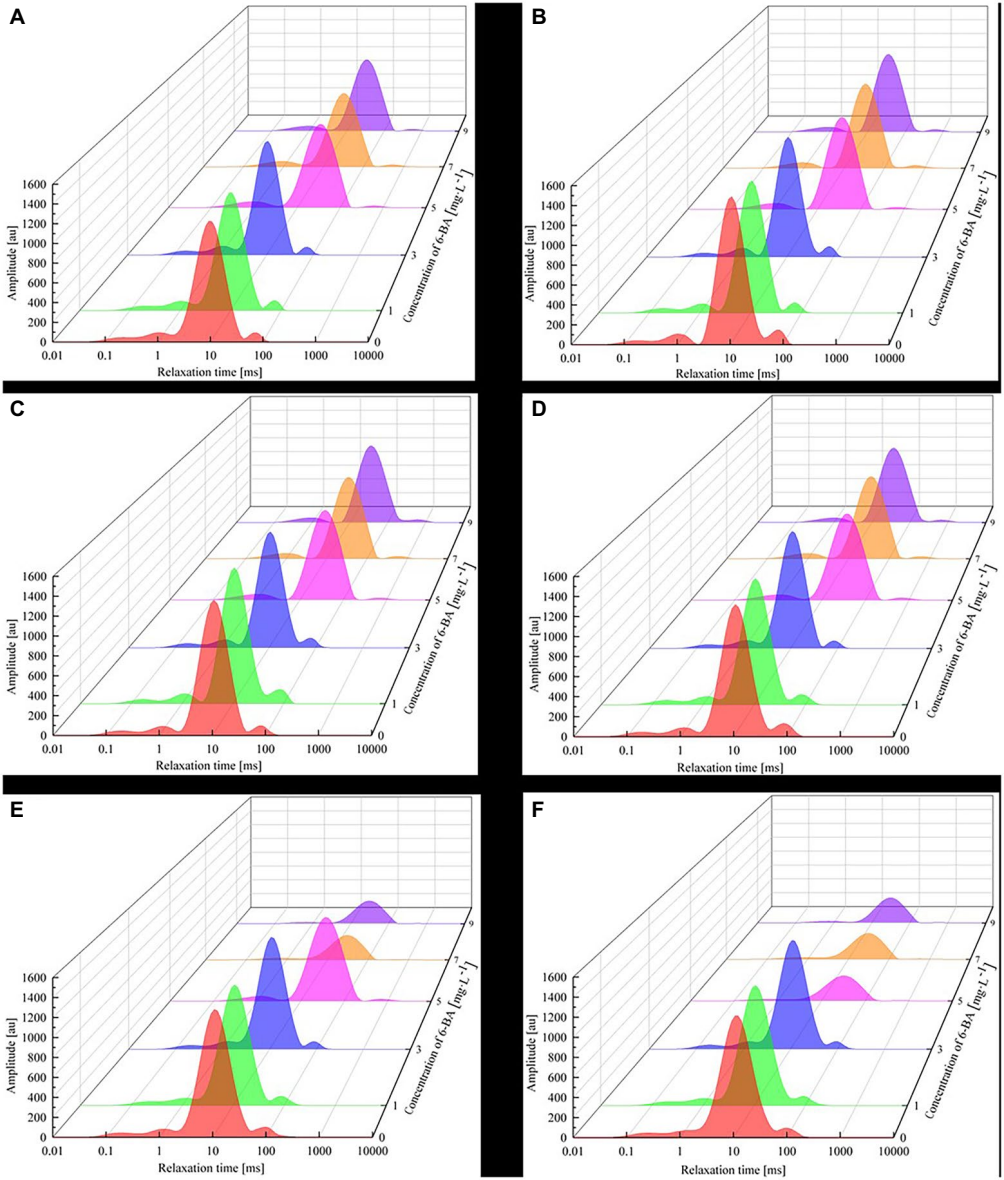
LF-NMR *T*_2_ relaxation time inversion spectrum waterfall of different germination times for different 6-BA concentrations: **(A)** soaking for 8 h, **(B)** germination for 12 h, **(C)** germination for 24 h, **(D)** germination for 36 h, **(E)** germination for 48 h, and **(F)** germination for 60 h. The experimental environment was as follows: temperature, 25°C; relative humidity, 50%; germination time, 0–60 h. The *T*_2_ relaxation time was determined using the CPMG sequence with the following parameters: SF, 21 MHz; SW, 200 kHz; TE, 0.25 μs; P1, 16 μs; P2, 36 μs; TW, 2000 μs; RFD, 0.02 ms; RG1, 20 db; DRG1, set to 3; and NECH, 3000.

Using the CPMG pulse sequence of a low-field nuclear magnetic resonance instrument, the CPMG echo peak map of the soybean samples was obtained, and the *T*_2_ inversion spectrum was obtained by determining the inversion of the echo peak points. To eliminate the influence of different quality soybeans on the results, Eq. (5) was used to normalize the *T*_2_ inversion spectra. Then, the *T*_2_ inversion spectrum waterfall of the germinating soybean was constructed for 0, 12, 24, 36, 48, and 60 h under different concentrations of 6-BA, as shown in Figure 4.

According to basic principles of NMR, the length of *T*_2_ can reflect the degree of water freedom in the sample; while a shorter *T*_2_ indicates lower water freedom in the sample, a longer *T*_2_ indicates the opposite (Song et al., 2016). Four clear peaks can be observed in the images presented in Figure 4, indicating the presence of four water populations: strongly bound water (*T*_21_): 0–1 ms; weakly bound water (*T*_22_): 1–10 ms; immobilized water (*T*_23_): 10–150 ms; and free water (*T*_24_): 150–10,000 ms (Lin et al., 2018). The corresponding signal amplitudes are *A*_21_, *A*_22_, *A*_23_, and *A*_24_, respectively. Moreover, the strongly bound water contains macromolecules, such as proteins and amino acids that decay rapidly. As such, their signals are difficult to capture by LF-NMR and are thus ignored. In addition, the internal oil content of soybeans overlaps with the weak combined water signal. Moreover, the soaking process has a negligible impact on the oil content of soybeans. In this study, the primary analysis focus was the internal water dynamics in soybeans; the oil signal of soybeans was not analyzed. Both strongly and weakly bound water molecules exist in soybean cells; they combine with proteins *via* strong hydrogen bonds. These water molecules cannot flow freely and are thus not involved in soybean metabolism. Moreover, the hydrogen bonding effect of strongly bound water molecules is notably greater than that of weakly bound water. Immobilized water combines with an amide (protein) and hydroxyl (starch and cellulose) group in the soybean. The hydrogen bond force in immobilized water is weak, and free water exists in the internal space within soybeans due to capillary action. Free water is characterized by significant fluidity, is a good solvent, can dissolve numerous substances and compounds, and participates in soybean metabolism. Moreover, the greater the free water content, the more vigorous the metabolic activity.

Water content has a major influence on seed germination. When soybean sprouts were cultured in low 6-BA concentration solutions (0, 1, and 3 mg/l), the total peak area gradually increased with increasing 6-BA concentration. Conversely, the total peak area of the soybean sprouts cultured in high 6-BA concentrations (5, 7, and 9 mg/l) gradually decreased with increasing 6-BA concentrations. According to the principle of LF-NMR, the peak area reflects the change in the sample water content, where a larger peak area denotes a greater water content. A low concentration level of 6-BA can increase cell wall plasticity, increase the cell volume, promote the biosynthesis of nucleic acids and proteins, increase new cytoplasmic components, and promote the longitudinal growth of cells (Garnczarska et al., 2010). Alternately, a high concentration of 6-BA can induce ethylene biosynthesis and inhibit elongation growth (Rajjou et al., 2012).

Figure 4 indicates that the relaxation time of the weakly bound water *T*_23_ increases at higher 6-BA concentrations (in Figure 4, the peak area widens, and the relaxation peak reverts to its original position). This is likely because, during the entire germination process in the low 6-BA concentration treatment, the soybean was protected and supported by the cell wall, which generated pressure and gradually maintained a dynamic balance inside and outside the cells. Therefore, the peak *A*_23_ of the relaxation spectrum maintained a slightly continuous growth state after soaking and imbibition. However, a higher 6-BA concentration level resulted in a higher intracellular solution than external concentration; after 12–36 h of germination, water molecules migrated from areas of low concentration to those of high concentration, and the peak *A*_23_ of the relaxation spectrum continued to increase. After germination for 48 h, soybeans germinated in the 7 and 9 mg/l 6-BA solutions were in a low concentration state compared to those cultured in the other 6-BA solutions. Therefore, the peak *A*_23_ of the relaxation spectrum decreased sharply. In addition, the high 6-BA concentration solutions destroyed the cell wall of the soybean, and the *A*_23_, *A*_21_, and *A*_24_ peaks gradually disappeared. After 60 h of germination, the soybean cultured in the 5 mg/l 6-BA solution also changed due to cell infiltration and damage to the soybean cell wall. At this stage, only some weakly bound water (*T*_23_) was detected in the soybean sprouts and moisture in other phases was not detected.

The population type of the water in the experimental sample was determined using LF-NMR experiments, and the effects of the various 6-BA concentrations on different water populations in the soybean were further analyzed.

### Effects of 6-BA on Different Phase Water in Soybean

Through the analysis of Table 1, it was found that under the treatment of different concentrations of 6-BA, the water content of each phase in soybean changed continuously with the increase of germination time. 6-BA had different effects on the water content of four phases in soybean: strong bound water, bound water, weakly bound water, and free water.

**Table 1:** Statistics table of unit mass *T*_2_ peak area.

Time/h	Concentration gradient (mg·L^−1^)	Strongly bound water *A*_21_	Weakly bound water *A*_22_	Immobilized water *A*_23_	Free water *A*_24_
0	0	186.5228 ± 19.1120a	24.9138 ± 2.2746a	925.5825 ± 29.0700a	0.9627 ± 0.0846a
	1	157.4211 ± 12.1709a	26.8403 ± 3.1749a	953.2339 ± 11.9085a	0.5970 ± 0.6393a
	3	178.4938 ± 10.3771a	27.0657 ± 2.7249a	1008.9495 ± 14.2126a	0.7008 ± 0.6649a
	5	177.6223 ± 7.3419a	26.9886 ± 2.5623a	915.5826 ± 12.3290a	0.5026 ± 0.1222a
	7	186.9986 ± 4.7909a	22.6522 ± 0.8466a	927.9406 ± 9.9926a	0.6748 ± 0.3080a
	9	176.1621 ± 19.5969a	24.9591 ± 2.1268a	901.5451 ± 10.9630a	0.5024 ± 0.1216a
8	0	404.5629 ± 41.6225b	443.4964 ± 27.0631ab	7546.7459 ± 149.9346 cd	177.1723 ± 24.3489a
	1	451.2651 ± 36.9420b	409.7575 ± 28.5437b	7358.6354 ± 215.8407d	123.7743 ± 21.6866b
	3	373.3993 ± 36.2162b	470.5863 ± 24.5296a	7729.0446 ± 96.1954bcd	181.0579 ± 20.9278a
	5	697.7455 ± 10.8796a	107.6300 ± 7.4889c	8328.3146 ± 110.3958a	0.7309 ± 0.3603c
	7	660.3819 ± 6.0550a	128.3319 ± 6.5745c	8175.1152 ± 104.3313ab	0.0000 ± 0.0000c
	9	622.4749 ± 11.6121a	122.3499 ± 7.8512c	7866.7514 ± 185.1568bc	0.0000 ± 0.0000c
12	0	379.2074 ± 41.7805b	520.1089 ± 23.3332a	8879.9191 ± 167.8829bc	290.1996 ± 39.5770a
	1	420.4034 ± 32.6581b	483.7990 ± 20.3180a	8416.6637 ± 188.0050d	199.5275 ± 26.7386b
	3	386.5081 ± 36.2351b	508.5109 ± 18.4805a	8565.7138 ± 139.5667 cd	292.6997 ± 27.9752a
	5	676.0009 ± 15.1759a	131.3725 ± 10.5501b	9213.8539 ± 100.9109ab	1.4429 ± 0.7679c
	7	683.1939 ± 11.7846a	182.0528 ± 25.1258b	9452.5231 ± 122.4345a	0.7093 ± 0.3853c
	9	633.2518 ± 10.2295a	174.4829 ± 11.6843b	8739.2547 ± 117.5928 cd	0.0000 ± 0.0000c
24	0	383.3038 ± 33.6641c	1071.3521 ± 397.5786a	8371.7184 ± 406.0183c	408.0388 ± 62.2276a
	1	364.7163 ± 28.7118c	503.0023 ± 20.2736b	8470.2079 ± 194.7436bc	305.4547 ± 38.1884b
	3	416.4235 ± 29.7795c	459.5273 ± 21.1729b	8727.1064 ± 133.5649bc	241.7669 ± 27.1374b
	5	693.5216 ± 17.5692a	129.9343 ± 12.8465b	9375.9785 ± 115.8796a	2.5809 ± 0.9189c
	7	700.5653 ± 14.3181a	197.7274 ± 21.9202b	9587.1300 ± 126.6174a	2.0874 ± 0.9232c
	9	609.1094 ± 11.5343b	193.6622 ± 14.8798b	9020.5223 ± 118.1884ab	0.6894 ± 0.3904c
36	0	445.9389 ± 33.6022a	503.1159 ± 52.8349ab	9111.0298 ± 189.7852ab	388.3361 ± 76.2503a
	1	401.5807 ± 32.3459a	451.2859 ± 17.9429ab	8594.0393 ± 183.8989b	301.7036 ± 46.1007ab
	3	669.5730 ± 296.8337a	727.9676 ± 310.3704a	7917.6990 ± 492.8833c	205.9596 ± 26.0099b
	5	663.9483 ± 15.9262a	160.8063 ± 9.8867b	9464.6155 ± 130.3771a	2.1699 ± 1.0492c
	7	672.7219 ± 14.3271a	154.5737 ± 13.5000b	9629.4817 ± 128.2051a	2.7593 ± 1.515c
	9	592.5212 ± 11.4241a	180.6471 ± 17.1772b	8993.2260 ± 125.9427ab	0.7858 ± 0.4419c
48	0	411.3442 ± 36.6574b	445.5939 ± 23.5706a	9314.5801 ± 193.1653ab	285.9690 ± 46.8754b
	1	401.1031 ± 29.0965b	346.4032 ± 28.2396b	8732.6681 ± 183.3153c	175.5920 ± 31.9143b
	3	416.2707 ± 47.6110b	383.5373 ± 25.9598b	8987.8238 ± 160.3275bc	1079.9779 ± 532.2952a
	5	589.8632 ± 31.4620a	151.6539 ± 12.3731c	9492.7169 ± 164.6214a	3.1225 ± 1.0358b
	7	220.9864 ± 14.9049c	31.2036 ± 2.6269d	3260.1613 ± 64.4901d	0.1537 ± 0.1537b
	9	205.2493 ± 8.0343c	37.6259 ± 2.3692d	2942.4345 ± 49.3086d	0.0050 ± 0.0001b
60	0	483.3020 ± 33.1773a	399.0594 ± 23.0401a	9735.5486 ± 176.5427a	105.0884 ± 34.0541b
	1	424.1696 ± 36.3614ab	388.7542 ± 23.5285a	9017.6594 ± 186.0608b	172.4321 ± 36.4174a
	3	379.4051 ± 27.2309b	421.7536 ± 24.8471a	8961.2632 ± 111.9489b	191.9567 ± 31.2203a
	5	218.1030 ± 8.6926c	30.7745 ± 2.5863b	3122.1812 ± 62.1478c	0.5089 ± 0.2114c
	7	265.5766 ± 16.9151c	33.8706 ± 2.9697b	3579.8205 ± 63.5494d	0.2131 ± 0.1499c
	9	225.6308 ± 9.5665c	39.6251 ± 2.5688b	3173.9110 ± 45.3145d	0.1187 ± 0.0854c

Strongly bound water peak area: A_21_, weakly bound water peak area: A_22_, immobilized water peak area: A_23_, free water peak area: A_24_. All statistical data were unit mass peak area, expressed by mean ± root mean squared error $\left(\bar{x}\pm s\right)$ . Different letters indicated that there was significant difference between different gradient groups (*P* < 0.0 5).

Soaking for 8 h is the imbibition stage of soybean. Water molecules enter soybean seeds through imbibition, and the water content increases rapidly, preparing for the later radicle growth. Under the action of different concentrations of 6-BA, there were significant differences between high concentrations (5, 7, 9 mg/l) and low concentrations (0, 1, 3 mg/l). Eight to twenty four hours is the germination stage. At this time, the rapid water absorption of soybean seeds in the imbibition stage leads to the saturation of protoplasm, the increase of cell turgor pressure, and the impediment of cell water absorption, so the content of water in each phase does not increase significantly. Twenty four to sixty hours was the germination stage of soybean, and the water absorption content of seeds gradually leveled off.

During the imbibition stage, soybeans swelled and germinated after absorbing enough water, and all water populations except free water content increased significantly. Overall, low- and high-concentration 6-BA treatments exhibited opposite hydrodynamic profiles. Under the action of low concentration of 6-BA, each peak changed slightly at 8–60 h. Under the action of high concentration of 6-BA, the contents of *A*_21_, *A*_22_, and *A*_23_ were greatly reduced at 60 h after germination, the average value of *A*_21_ dropped sharply below 300, the average value of *A*_22_ dropped sharply below 50, and the average value of *A*_23_ dropped sharply below 4,000. The variation trends of 6-BA concentration gradients on soybean total moisture *A*_24_ are all shown as follows: under the action of high concentration 6-BA, the free water *A*_24_ content has a decreasing trend, and the reason for the reduction is that the moisture of each phase can interact with each other to a certain extent. Transformation, during germination, high concentration of 6-BA accelerates the mobility of water between phases. Under the action of low concentration of 6-BA, the content of *A*_24_ in free water showed a regular change of increasing–smoothing–decreasing.

Throughout the germination process, complex exchanges between moisture components occur. 6-BA can decompose nutrients such as carbohydrates and proteins stored in seeds into small molecular substances, which can be absorbed and utilized by the germ, breaking seed dormancy and promoting seed germination. Soybean seeds are rich in plant protein. 6-BA affects soybean seed water absorption by affecting the protein body of soybean seeds. During the germination process, the protein is gradually decomposed along with the swelling of the protein body and the change of the storage tissue.

### Analysis of PCA Results

**Figure 5:**
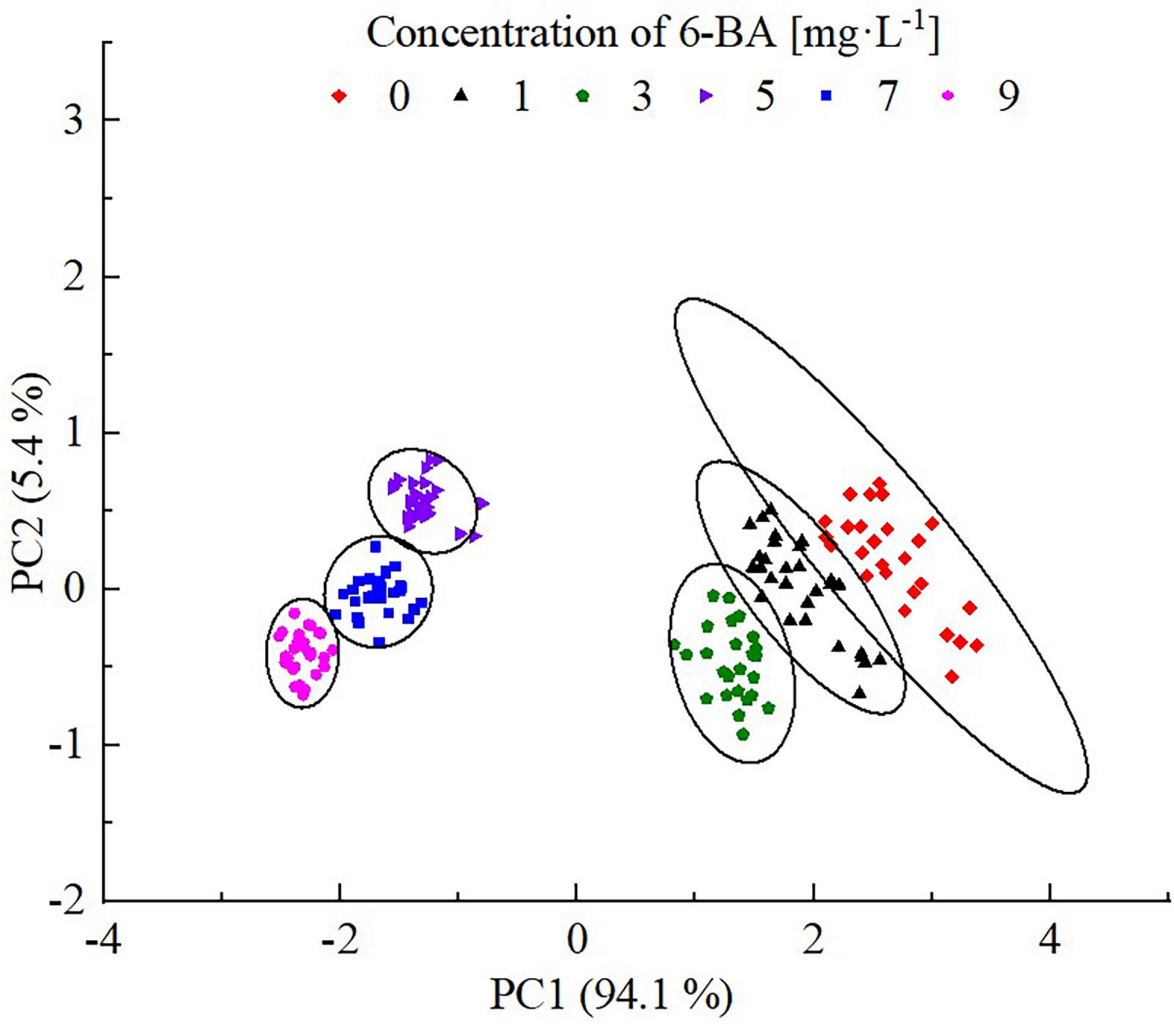
PCA scores of soybean sprouts cultured with different concentrations of 6-BA. Each dot represents a sample; each color represents a 6-BA concentration.

The spectral data of the soybean sprouts cultured in the 6-BA solutions were collected, and the CPMG back peak data were inverted and normalized. The peak point data of the CPMG detected for the sample were listed as multiple variables in a row; the row vector represented the experimental sample. The peak area data of the soybean sprouts treated with different concentrations of 6-BA were combined to form the original data matrix. Soybean sprouts cultured with different concentrations of 6-BA were detected for adulteration by principal component analysis, as shown in Figure 5. Each point in Figure 5 represents the soybean sprout samples. An area of the same color and shape represents the overall quality difference of the experimental sample. The image is divided into six areas to represent the characterization of soybean sprouts cultured in different 6-BA concentrations.

In Figure 5, principal component 1 and principal component 2 show the contribution rates of each component obtained from the PCA conversion. The greater the contribution rate, the more information could be reflected based on the indicators of the original data. The contribution rate of principal component 1 was 94.1%, and that of principal component 2 was 5.4%. The two principal components retained 99.5% of the original data. The experimental samples in the same area represent the same processing methods, that is, parallel samples. The processing effect of different samples could be characterized by the interval distance in the figure: the greater the interval, the more obvious the effect. With the increase in 6-BA concentration, the distribution of soybean samples shifted from right to left on the principal component 1 axis, showing a certain regularity: the distribution of samples with 6-BA concentrations of 0, 1, and 3 mg/l overlapped partially, and the distribution distance of samples with 6-BA concentrations of 5, 7, and 9 mg/l was relatively dense. This shows that the quality of soybean sprouts treated with low concentrations and high concentrations of 6-BA clearly differed, further demonstrating the rationality of the model.

### Different Detection Techniques Are Compared

High demand for quality and safe processed foods has stimulated the quest in innovative technologies in food science owing to the shortcomings of the existing or conventional technologies with respect to their sensitivity, selectivity, robustness, cost-effect and timeliness (Ezeanaka et al., 2019). To overcome these challenges, alternative techniques such as near-infrared spectroscopy (NIR), Raman spectroscopy, hyperspectral imaging (HSI), and NMR used in food quality analysis are used nowadays. They provide accurate results when compared with the conventional or traditional techniques. Raman spectroscopy can be used for both liquid and solid samples, which can provide detailed information about molecular vibration related to secondary and tertiary structures of protein, lipid structures, and water structures (Velioğlu et al., 2015; Zhang et al., 2020). NIR spectral information is derived from the double frequency and combined frequency absorption of organic hydrogen-containing groups (C-H, O-H, N-H), free water signal from frozen samples is easier to detect with NIR (Liu et al., 2020). HSI is a technology that combines vision technology and Vis–NIR spectroscopy, which allows the acquisition of spectral and spatial information simultaneously. The potential nature of non-destructive, cost-effective, and rapid sensing procedures is widely accepted in the fresh produce industry for quality assessment and detection of storage and quality issues (Pullanagari and Li, 2020). The major advantage with NMR is that it is both robust and non-destructive to the sample and require no sample treatment prior to analysis.

However, these techniques have some limitations in food process control monitoring. For instance, NIR not only needs some calibration models to be established mostly for online application but also is not reliable and sufficiently steady when used. For Raman spectroscopy, high cost and non-stability of the instrument limit its application in process analysis. The complex mechanism of hyperspectral imaging and the huge amount of data make it difficult to preprocess image data (Ezeanaka et al., 2019). NMR is a fundamental analytical technique that can obtain specific information about the proteins, lipids, and water; however, this technique is not suitable for detecting minor components such as low molecular weight proteins (Zhang et al., 2020). Therefore, further research and development are needed to counter these drawbacks. In-depth mining of information features, reducing the cost of constructing prior knowledge, and realizing the transition from feasibility to practicality will be the further research direction of non-destructive testing technology.

## Conclusion

NMR proton density pseudo-color maps obtained by MRI experiments upon soybeans treated with different concentrations of 6-BA at continuous time points were constructed to assess the soybean quality and measure the transverse and longitudinal axis diameters of soybeans. The analysis of the gray value of the ROI and the changes in soybean quality and volume showed that a low 6-BA concentration promoted water absorption and soybean germination, whereas high concentrations produced inhibitory effects. In this experiment, the water content of the soybean was highest when the culture concentration of 6-BA was 5 mg/l. Moreover, four types of water populations were detected in the soybean: strongly bound water (*T*_21_), weakly bound water (*T*_22_), immobilized water (*T*_23_), and free water (*T*_24_). With an increase in the 6-BA solution concentration, the proportions of the four types of water and the relaxation time were uniquely altered. In addition, PCA analysis could effectively distinguish the concentration of the 6-BA solution containing the soaked soybean with relatively high accuracy. Moreover, the experimental soybean samples showed a regular distribution with an increase in the 6-BA concentration. At present, the literature mostly focuses on the time-dependent growth dynamics of soybean, and rarely a spatio-temporal analysis has been attempted. Furthermore, only a few studies have previously reported the effect of 6-BA on water dynamics during soybean sprout growth in China or other countries.

In conclusion, LF-NMR/MRI can reflect the post-germination water absorption and distribution in soybean seeds thanks to its non-invasive and non-destructive characteristics. Non-destructive testing technology for agricultural products is the basic type of technology for testing the quality of agricultural products.

## Data Availability Statement

The raw data supporting the conclusions of this article will be made available by the authors, without undue reservation.

## Author Contributions

YG designed the experiments, analyzed the data, and wrote the manuscript. YC and XY finished all most experiments. PS and BL revised the manuscript. The others participated in experiments or manuscript. All authors contributed to the article and approved the submitted version.

## Funding

The authors acknowledge the support of the National Natural Science Foundation of China (31701318, 31811540396) and the basic research project of Liaoning Province (LSNJC201916).

## Conflict of Interest

The authors declare that the research was conducted in the absence of any commercial or financial relationships that could be construed as a potential conflict of interest.

## Publisher’s Note

All claims expressed in this article are solely those of the authors and do not necessarily represent those of their affiliated organizations, or those of the publisher, the editors and the reviewers. Any product that may be evaluated in this article, or claim that may be made by its manufacturer, is not guaranteed or endorsed by the publisher.

## Abbreviations

LF-NMR: low-field nuclear magnetic resonance
RH: relative humidity
NMR, nuclear magnetic resonance.
*T*_2_: transverse relaxation times
CPMG: Carr-Purcell-Meiboom-Gill
SF: corresponding resonance frequency
SW: spectral width
TE: echo time
TR: repeat sampling time
RF: radio frequency
P1: pulse widths of 90°
P2: pulse widths of 180°
TW: waiting time
RFD: Rf delay time
RG1: analog gain
DRG1: digital gain
6-BA: 6-benzylaminopurine
PCA: principal component analysis
ROI: region of interest
FOV: field of view
OR: offset read
